# Polyphenols: Natural food grade biomolecules for treating neurodegenerative diseases from a multi-target perspective

**DOI:** 10.3389/fnut.2023.1139558

**Published:** 2023-02-28

**Authors:** Zhenmin Li, Ting Zhao, Mingqin Shi, Yuanyuan Wei, Xiaoyi Huang, Jiayan Shen, Xiaoyu Zhang, Zhaohu Xie, Peidong Huang, Kai Yuan, Zhaofu Li, Ning Li, Dongdong Qin

**Affiliations:** ^1^School of Basic Medical Sciences, Yunnan University of Chinese Medicine, Kunming, China; ^2^The First Clinical Medical School, Yunnan University of Chinese Medicine, Kunming, Yunnan, China; ^3^The Second Clinical Medical School, Yunnan University of Chinese Medicine, Kunming, Yunnan, China

**Keywords:** polyphenols, antioxidant, anti-inflammation, neurodegenerative diseases, efficacy, mechanism

## Abstract

As natural functional bioactive ingredients found in foods and plants, polyphenols play various antioxidant and anti-inflammatory roles to prevent the development of disease and restore human health. The multi-target modulation of polyphenols provides a novel practical therapeutic strategy for neurodegenerative diseases that are difficult to treat with traditional drugs like glutathione and cholinesterase inhibitors. This review mainly focuses on the efficacy of polyphenols on ischemic stroke, Parkinson's disease and Alzheimer's disease, including *in vivo* and *in vitro* experimental studies. It is further emphasized that polyphenols exert neuroprotective effects primarily through inhibiting production of oxidative stress and inflammatory cytokines, which may be the underlying mechanism. However, polyphenols are still rarely used as medicines to treat neurodegenerative diseases. Due to the lack of clinical trials, the mechanism of polyphenols is still in the stage of insufficient exploration. Future large-scale multi-center randomized controlled trials and in-depth mechanism studies are still needed to fully assess the safety, efficacy and side effects of polyphenols.

## Introduction

Neurodegenerative diseases are a heterogeneous group of diseases characterized by irreversible, progressive degeneration and death of neuronal cells (1). At present, the cause of this kind of disease is still unclear and cannot completely cured yet, which poses a severe challenge to human health and a substantial economic burden. Neurons present in the body can maintain cellular homeostasis by dealing with different stressors (2). However, two conditions usually lead to neuronal death. The first one is when multiple stressors accumulate and exceed the cell's ability to recover, various damages to cells will eventually lead to neuronal death. The formation of higher-order aggregates is a significant cause of neuronal stressor, and eventually triggers consequent cytotoxic events and cell death (3–6). Another cause of neuronal death is the traumatic events, such as ischemic stroke, which usually leads to a massive decrease in neuronal function in the affected area and subsequently induces acute neuronal cell death (7). In recent years, the improvement of neurodegenerative diseases through natural medicines such as polyphenols has increasingly become a research hotspot.

Polyphenols are secondary plant metabolites with highly diversified chemical structures (8). They are the largest class of plant chemicals. The basic structure of polyphenols is to connect at least one aromatic ring with one or more hydroxyl functional groups (i.e., several hydroxyl groups on the aromatic ring). Thousands of such polyphenol structures have been identified in plants and foods, mainly including flavonoids (60%), phenolic acids (30%), and other polyphenols, such as stilbene and lignans (8, 9). Flavonoids can be divided into anthocyanins, flavan-3-oil, flavonoids, flavanone, and other flavonoid subclasses. Phenolic acid exists in free form in fruits and vegetables, and they often exist in a conjugated state in bran and shell (10–12). Wine and red wine contain stilbene in other polyphenols, and lignans are found in many grains, such as sesame (13, 14). Polyphenols, as a powerful antioxidant, can play an essential role in the treatment of oxidative stress and neuroinflammation-related diseases. It has been found that supplementing antioxidant vitamins and enzymes, such as vitamins C, E, carotene, and other antioxidants, can protect the organism against external stimuli, reduce and eliminate the level of reactive oxygen species (ROS) (14, 15). Recently, the side effects of traditional drugs used to treat neurodegenerative diseases, such as cholinesterase inhibitors and NMDA antagonists, have become more widely recognized (16). Compared to other drugs, polyphenolic natural medicines are often found in our daily diet, and they have fewer side effects. Many plant foods that are common in our daily lives contain polyphenols, such as tea, cocoa, fruits, and vegetables. In addition to this, polyphenols have also been found in traditional Chinese medicine (17).

The preventive and therapeutic effects of polyphenols on neurodegenerative diseases have been investigated in previous studies (18–20). Although there are relatively many studies on polyphenols, the underlying core mechanism of polyphenols in neurodegenerative diseases is still unclear. This review will focus on the effects of polyphenols on neurodegenerative diseases, including cerebral ischemic stroke (CIS), Parkinson's disease (PD), and Alzheimer's disease (AD), which covers both *in vitro* and *in vivo* studies. Furthermore, the underlying mechanisms that polyphenols exert neuroprotective effects are also reviewed.

## Effects and mechanisms of polyphenols in the treatment of neurodegenerative diseases

**Figure 1:**
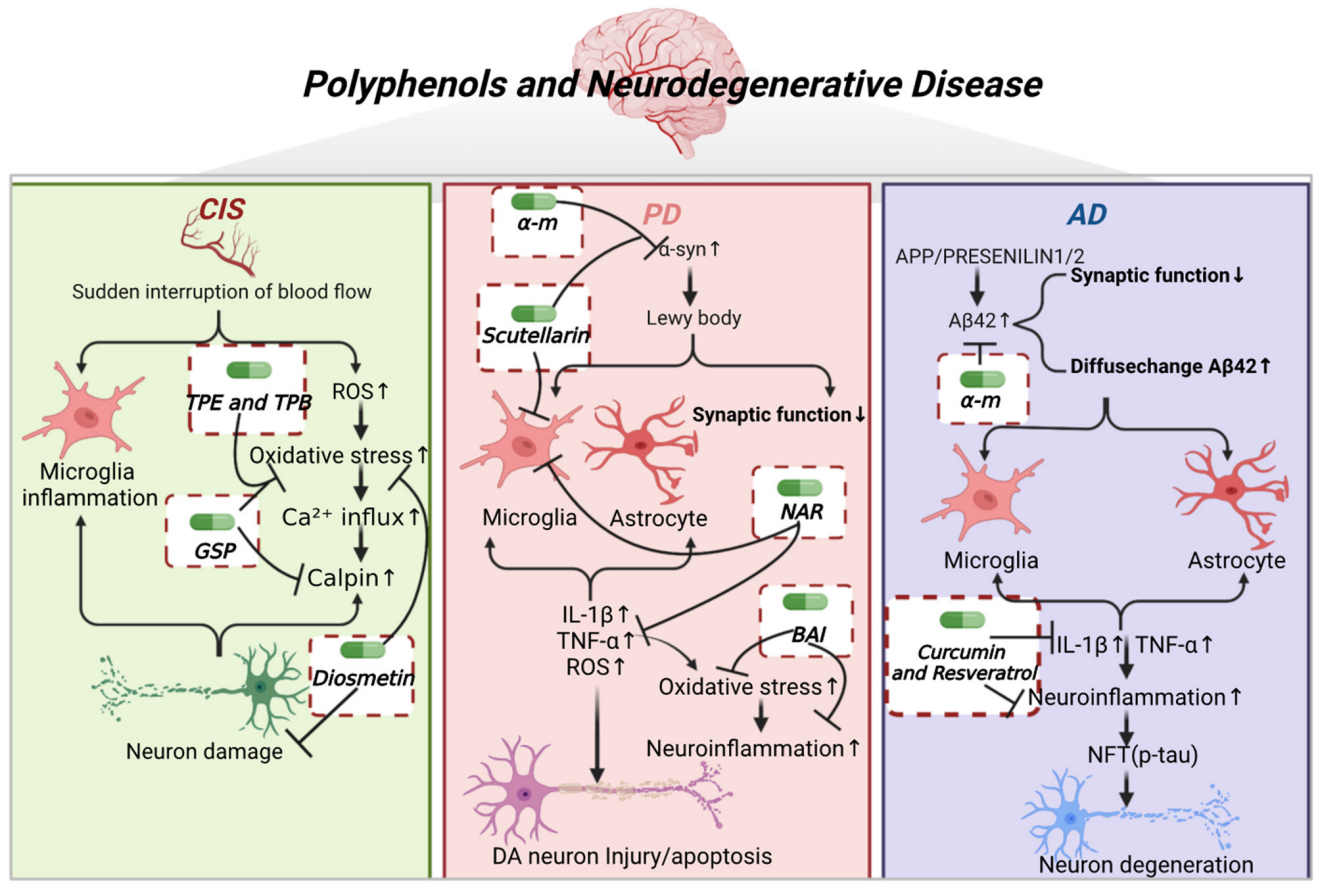
Polyphenols and neurodegenerative diseases. CIS is caused by the sudden interruption of blood flow to the brain, leading to brain cell death and nerve damage. Subsequently, it causes microglia activation, elevates ROS, inward calcium flow, and calmodulin release, leading to neuronal death. TPE, TPB, GSP, and diosmetin inhibited oxidative stress. GSP also inhibited calpain activation, and diosmetin exerted neuroprotective effects. PD is caused by the aggregation of α-syn proteins into Lewy bodies, leading to decreased synaptic function, and further activation of microglia and astrocytes. This leads to the release of inflammatory factors such as IL-β, TNF-α, and ROS, causing elevated levels of oxidative stress, neuroinflammation and damage or apoptosis of DA neurons. α-M and scutellarin inhibited the aggregation of α-syn oligomers. Scutellarin and NAR inhibited the activation of microglia. NAR inhibited the release of inflammatory factors. BAI inhibited the oxidative stress and neuroinflammation. AD is caused by the deposition of Aβ42 aggregates, which activates microglia and astrocytes to release inflammatory factors and leads to neuroinflammation as well as NFT. α-M inhibited the aggregation of Aβ42 oligomers. Curcumin and resveratrol inhibited the release of inflammatory factors and neuroinflammation, thereby attenuating neuronal degeneration.

As shown in Figure 1, the pathogenesis of neurodegenerative diseases, as well as the core target and underlying mechanism of polyphenols in the treatment of neurodegenerative diseases are described, including cerebral ischemic stroke (CIS), Parkinson's disease (PD), and Alzheimer's disease (AD). Table 1 further illustrates the type of polyphenols, and the effects and mechanism *in vitro* and *in vivo* experiments.

**Table 1:** Effects and mechanisms of polyphenols in the treatment of neurodegenerative diseases.

**Compounds**	**Disease**	**Model**		**Effects and mechanisms**		**References**
		***In vitro***	***In vitro***	***In vitro***	***In vitro***	
TPE and TPB	CIS	BV2 microglial cells	Mice: MCAO	Facilitated the translocation of Nrf2 to the nucleus and enhanced Nrf2 expression in the nucleus and restored OGD/R-induced oxidative damage	Alleviate the reduction of antioxidant enzyme activity in cerebral ischemia-reperfusion injury, exerted protective effects in MCAO mice by inhibiting apoptosis, and stimulating the Nrf2/HO-1 signaling pathway	(21)
Geraniin		PC12 cells	SD rats: MCAO/R	Protect PC12 cells from OGD/R-induced cytotoxicity and oxidative stress, protect PC12 cells from OGD/R-induced cell apoptosis, activate of the Nrf2/HO-1 signaling pathway *in vivo* and *in vitro*	Protects against I/R injury, suppresses oxidative stress induced by MCAO/R, attenuates MCAO/R-induced neuronal apoptosis *in vivo*	(22)
GSP		Primary brain Neuron-astrocyte cell: OGD/R	Wistar rats: MCAO	GSP improved cell viability, exerted a powerful anti-inflammatory effect as it counteracted OGD-induced pro-inflammatory cytokines expression or anti-inflammatory cytokines expression	GSP-induced calpain activity inhibition or enhancement of neurotrophic factors such as BDNF	(22)
Resveratrol		Neurons: OGD/R	SD rats: MCAO	Inhibit the decrease of cell viability and apoptosis induced by OGD/R, and activate the mitochondrial phagocytosis induced by OGD/R, improve the recovery of motor function.	Improve the recovery of motor function	(23, 24)
Scutellarin	PD	α-Syn fibrillation: Fe^3+^-and Al^3+^ induced		Inhibited the activation of microglia and inhibited the release of inflammatory factors		(25)
CGA		Enteroendocrine L Cells	Male C57BL/6 J mice	Increased intracellular cAMP levels, increased the release of GLP-1 and GLP-1 release	Improves rotenone- induced behavioral and cognitive deficits in mice, recovers rotenone-induced oxidative damage to the striatum and cortex of Parkinson's disease	(26)
NAR		BV-2 cells	SD rats	Inhibited the Activation of Microglia and NLRP3 Inflammasome	It can reduce the loss of LPS-induced DA neurons	(27)
α-Mangostin (α-M)		PC12 cell: rotenone-induced	Male C57BL/6J mice: rotenone-induced	Promotes autophagy-directed clearance of α-Syn majorly through the activation of AMPK	It inhibits oxidative stress in the cortex, improves rotenone-induced behavioral defects in mice treated with rotenone, alleviates rotenone-induced striatal DA ergic neuron degeneration and rotenone-treated SNc in mice, and alleviates rotenone-induced α-Syn accumulation	(28)
Procyandin A2 (PCA2)	AD	RAW264.7: LPS stimulation	Mice: D-galactose-induced aging mice	Anti-inflammatory and antioxidant effects	Down-regulate NLRP3 inflammatory body signal pathway to inhibit inflammation in brain tissue	(10, 29)
Curcumin		PC12 cell: Aβ stimulation	Rats: damaged by H2O2	The neuroprotective effect, lower Aβ Oligomer induced neurotoxicity, reducing LPS- induced neuroinflammation	Anti-oxidative properties of AM, reduce senile plaque and repair neuron damage	(30, 31)
Baicalein		Neuroblast: Exposure to Aβ Oxidative stress model of neuroblastoma SH-SY5Y cell line	APP/PS-1 double transgenic mice	Reduce oxidative stress and neuroinflammation and protect nerves. Reduced ROS production, reduced oxidative stress, inhibited tau hyperphosphorylation, and protected SH-SY5Y cells from Aβ O Damage	Effectively improve memory impairment and restore cognitive function	(32)
Trehalose		HAW cells: overexpress the APP695 gene 20E2 cells: overexpress the APP695 gene	Mice: Bilateral intraventricular injection Aβ fragment.	It affects App Processing and Decreases Aβ	Blocked Aβ Deposition and microglial activation	(33)
Resveratrol		PC12 cells: Add Aβ_1 − 42_ to the culture medium induction.	Mice: SIRT1Dex4/Nestin-Cre mice Rats: Ovariectomized (OVX) + d-galactose (d-gal)	Prevent neuronal damage, inhibit Aβ_1 − 42_, Induces apoptosis, reduces oxidative status, and alleviate mitochondrial damage	Improve the impaired learning and memory in neurodegenerative diseases, and protect the memory decline in AD through its antioxidant activity	(34)

### Cerebral ischemic stroke

Cerebral ischemic stroke is one of the leading causes of disability in the world's population. CIS is caused by the sudden interruption of blood flow to the brain, leading to brain cell death and neuronal damage (35). Because of the poor regenerative capacity of the adult brain, neuronal damage is almost impossible to reverse (36). Therefore, it is essential to reduce the loss of neurons or tissues after CIS (37). Cerebral ischemia/reperfusion (I/R) injury is an important risk factor for stroke because the process of I/R may further aggravate the initial ischemic injury. For this reason, the pathogenesis of CIS has not been fully clarified, and there is a lack of effective treatments (38).

### *In vitro* studies

Oxidative stress caused by excessive ROS production is closely related to the pathogenesis of CIS. At the same time, oxidative stress is one of the most important processes in brain I/R injury and the main risk factor for neural cell apoptosis (39). Excessive ROS production leads to neuronal dysfunction through a variety of mechanisms, including inflammation, cell apoptosis and necrosis (40), which is considered to be the main factor leading to brain damage (41, 42). Resveratrol is a natural plant antitoxin, which has neuroprotective, antioxidant, anti-cancer, and anti-inflammatory properties (43–45). A study has found that resveratrol exerts its protective effects against the damage of oxygen glucose deprivation and reperfusion (OGD/R), at least in part, by promoting mitophagy (23). Resveratrol attenuates OGD/R-induced oxidative stress and preserves mitochondrial function, exerting neuroprotective effects through PINK1/Parkin-mediated mitophagy. Grape seed proanthocyanidins (GSP) are one of the complex flavonoid polymers. Many studies show that GSP have anti-inflammatory, anti-apoptotic, antioxidant, and free radical scavenging properties (29, 46, 47). One study established an OGD/R model using primary brain cell cultures (48). They found that GSP could prevent the damages of OGD/R, inhibit OGD/R-induced cell death and improve cell viability. Pretreatment with GSP can efficiently prevent the cells from almost all inflammatory factors. Another experiment identified the main biological activity of TPE and TPB by high performance liquid chromatography (HPLC) as phenolic acids after grading and purification of crude extracts (21). It was found that TPE and TPB facilitated the translocation of nuclear factor erythroid-2 related factor 2 (Nrf2) to the nucleus and enhanced Nrf2 expression in the nucleus, restored OGD/R-induced oxidative damage in BV2 microglial cells. Pelargonium is a polyphenol extracted from phyllanthus amarus, which has a wide range of biological and pharmaceutical activities, including antioxidant, anti-inflammatory, antithrombotic, and other biological activities. In addition, pelargonium has been found to activate the nuclear factor erythroid-2 related factor 2/heme oxygenase-1 (Nrf2/HO-1) signaling pathway and protect PC12 cells from cytotoxicity, as well as oxidative stress and apoptosis caused by OGD/R (22).

### *In vivo* studies

Two-month-old male SD rats were modeled by middle cerebral artery occlusion (MACO) (24), and rehabilitation training plus resveratrol was used for treatment. It was found that resveratrol improved the recovery of neurological and motor function in MCAO rats through the silent mating type information regulation 2 homolog-1 (Sirt1) signaling pathway, and activated brain-derived neurotrophic factor/tyrosine kinase receptor b (BDNF/TrkB) signaling pathways. By modeling the I/R injury of male Wistar rats (48), it was found that the treatment of GSP reduced the size of cerebral infarct and clearly improved both behavioral and overall activities of rats as it allowed to restore rearing and crossing scores to near control level. Another study demonstrated that the TPE and TPB alleviated the reduction of antioxidant enzyme activity in cerebral ischemia-reperfusion injury, and exerted protective effects in MCAO mice by inhibiting apoptosis and stimulating the Nrf2/HO-1 signaling pathway (21). Geraniin can reduce brain I/R damage by inhibiting oxidative stress (22). Geraniin can activate Nrf2/HO-1 signal pathway, reduce I/R damage of the middle cerebral artery occlusion-reperfusion (MCAO/R) in rats, significantly increase the number of surviving neurons, and inhibit oxidative stress induced by MCAO/R.

Therefore, polyphenols can inhibit oxidative stress to prevent neurons against the damage of ROS, exerting neuroprotective effects on CIS.

### Parkinson's disease

Parkinson's disease (PD), a progressive motor dysfunction, is defined as a significant loss and misfolding of dopaminergic (DA) neurons in the substantia nigra (SN), α-Synuclein (α-Syn) aggregation in louis corpuscles, as well as motor dysfunction (static tremor, rigidity, and motor retardation) and non-motor symptoms (autonomic nervous dysfunction, cognitive impairment, depression, REM sleep behavior, etc.) that appears several years before the onset of the motor phenotype (49–54). Studies have found that most PD patients are sporadic, and the rest are mostly related to gene mutations caused by mitochondrial dysfunction. About 50% of early-onset PD patients have mutations in Parkin (55–57).

### *In vitro* studies

α-Syn is one of the first genes found to be associated with PD. Research proves that α-Syn and Nrf2 deficiency aggravates protein aggregation, neuroinflammation, and neuronal death (58). A study has found that scutellarin can effectively inhibit metal-induced and uninduced α-Syn's fibrosis (25), and stabilize partially folded α-Syn intermediate to form an SDS-resistant high-order oligomer. Baicalin (BAI) is an important flavonoid compound. Another study by human cell line pLVX-Tet3G-α-synuclein SH-SY5Y found that BAI could protect DA neurons against ROS and decrease C/EBPβ and α-Syn expression in pLVX-Tet3G-α-synuclein SH-SY5Y cells (59). Alpha-mangostin (AM), a polyphenolic xanthone obtained from Garcinia Mangostana L, can activate the autophagy in PC12 cells, playing roles in clearance of α-Syn (28). Chlorogenic acid (CGA) is a polyphenolic compound with antioxidant and anti-inflammatory properties. CGA initiated the SIRT1/NF-κB signaling pathway and inhibited OGD/R-induced inflammation, oxidative stress, and neuronal apoptosis by upregulating MIR497HG to suppress miR-29b-3p expression (60). Naringin (NAR) is a natural flavone contained in citrus fruits and grapefruit, which has a lot of pharmacological activities. A study has proved that NAR protects DA neurons from lipopolysaccharide (LPS)-induced neurotoxicity by inhibiting the activation of inflammatory corpuscle signals of microglial NOD-like receptor pyrin domain containing 3 (NLRP3) and the subsequent release of proinflammatory factors (27).

### *In vivo* studies

A study of rotenone-induced male C57BL/6J mice proved that AM improved the behavioral deficiency induced by rotenone (28), and offset the oxidative stress in striatum and cortex, and decreased the degradation of DA neurons. DA can regulate innate immunity and inhibit systemic inflammation through different subtypes of dopamine receptor (DR) (61). DA also inhibits neuroinflammation in the brain through astrocyte DR2 and downstream signal transduction (62). Neuroinflammation is related to DA neurodegeneration and is a critical factor in the pathogenesis and progression of PD (63–66). A study modeled adult male C57BL/6 mice using LPS (67), and found that BAI could inhibit the activation of hippocampal glial cells and cytokine release, inhibit SIRT1 and downregulate the expression of high mobility group protein 1 (HMGB1) in microglial cells, preventing LPS-induced cognitive dysfunction and neuroinflammation, and producing neuroprotective effects. Another study modeled the male C57BL/6 mice *via* 1-methyl-4-phenyl-1,2,3,6-tetrahydropyridine (MPTP) (59), and found BAI protected dopaminergic neurons and rescued motor dysfunction. Chlorogenic acid (CGA) could downregulate rotenone-induced phosphorylated α-Syn levels by upregulating PI3K/AKT signaling pathway and inactivating GSK-3β *via* GLP-1, which improved rotenone-induced dopaminergic neurodegeneration and α-Syn accumulation in the substantia nigra, and enhanced striatal dopaminergic mean density of nerve fibers and eventually prevented rotenone-induced motor and cognitive impairments (26). NAR regulates PQ-induced DRD2, DAT, LRRK2, SNCA, β-linked protein, cystathionine-3 and BDNF genes, alleviates the loss of dopaminergic neurons (68). By modeling adult male Sprague-Dawley rats through LPS (27), it has been shown that NAR ameliorates LPS-induced decrease in TH protein expression and inhibits the activation of microglia and NLRP3 inflammasome, thus protecting DA neurons.

Therefore, it has been evidenced that polyphenols can disintegrate upstream α-Syn aggregation and inhibit the further development of PD. Moreover, polyphenols can also inhibit inflammation, reduce oxidative stress, play a neuroprotective role in regulating downstream events to protect DA neurons, and hinder the progression of PD.

### Alzheimer's disease

AD is a neurodegenerative disease, which is the most common type of dementia. AD can lead to progressive cognitive decline, irreversible loss of memory and cognitive function, and interfere with daily activities. Its main characteristics are Aβ deposition of aggregates in extracellular amyloid plaques (senile plaques), followed by degenerated neurons containing neurofibrillary tangles (NFT), which are mainly composed of hyperphosphorylated microtubule-associated protein Tau and synaptic loss (1, 2, 69–73).

### *In vitro* studies

The occurrence of AD is closely related to neuroinflammation. Some studies have found that neuroinflammation and microglia activation exist in the early stage of AD (74). In the AD model, microglia and astrocytes have been proven to produce various proinflammatory cytokines (3–5, 75). Resveratrol is a polyphenol commonly found in grape skins (Vitaceae). More and more studies have shown that resveratrol has anti-inflammatory, antioxidant, and anti-diabetes effects and can improve cognitive decline. Resveratrol has been proven to exert inhibitory activity on neuroinflammation and can inhibit the release of proinflammatory factors from microglia and astrocytes (76–78). Microglia can eliminate Aβ sediment and activate phagocytosis to restore tissue homeostasis (79, 80). However, when activation becomes chronic, microglia release excessive cytotoxic mediators, including proinflammatory cytokines, chemokines, complement components, ROS, and nitrogen species, which will cause neuronal cell degeneration (81, 82). Curcumin was first isolated from Curcuma longa L. in 1870 as a low molecular weight polyphenol compound. Studies have shown that curcumin has many beneficial pharmacological effects, including anti-cancer, anti-virus, anti-arthritis, anti-oxidative stress, anti-inflammatory, and neuroprotective properties (83). Inhibiting inflammation-promoting factors released by microglia are important targets of curcumin in treating AD. It has been shown that curcumin and its analogs cur 6 and cur 16 inhibit the secretion of pro-inflammatory mediators IL-1β and TNF-α released by microglia after stimulation of HMW Aβ_42Os_ (32). The senile plaque can induce AD, and the “amyloid cascade hypothesis” is the most accepted hypothesis of AD etiology (2). Aβ_42_ oligomer is a soluble and diffusible Aβ species that play a key role in synaptic loss and synaptic damage in individuals with mild cognitive decline and can further trigger synaptotoxicity and neurotoxicity, glial cell proliferation, and activation, inflammation, or cell death (3–7, 84). Aβ also has the ability to bind to pattern recognition receptors on glial cells (including astrocytes and microglia), and it contributes to the progression and severity of AD (32, 85, 86). α-Mangostin (α-M) is a kind of polyphenol flavone from polygonatumodoratum, which has been proven to be effective for clearness of Aβ. It was found that α-M favored Aβ fiber generation, decomposition, uptake, and degradation, which reduced Aβ neurotoxicity induced by oligomer and prevented LPS-induced neuroinflammation (30).

### *In vivo* studies

A study has investigated 90 AD patients by using a randomized double-blind method (87). Compared with the donepezil hydrochloride group, the resveratrol (RES) group achieved a significantly higher efficacy rate, MMSE score and FIM score, and the clinical indicators and ADAS cog score were significantly lower. There was no significant difference in the total incidence of adverse reactions. It proves that polyphenols have obvious effects on the treatment of AD diseases, including improvement of the inflammatory factor, and promotion of the cognitive function and prognosis (87).

Thus, polyphenols inhibit the formation and degradation of senile plaques in AD, thereby protecting the function of synapses. In addition, polyphenols play a role in inhibiting the release of inflammatory factors and neuroinflammation in AD, preventing the formation of NFTs and neuronal death, thus inhibiting the progression of AD.

## Perspectives and conclusion

Polyphenols show a significant neuroprotective effect, but the lack of understanding of the underlying mechanism limits its clinical application. This minireview makes a comprehensive investigation to reveal the role and mechanism of polyphenols in the treatment of neurodegenerative diseases, including *in vitro* and *in vivo* studies. It has been found that polyphenols, as multi-target drugs, play a crucial role in inhibiting the formation of pathological products, such as oxidative stress and inflammatory factors. Polyphenols have two advantages as drugs for the treatment of neurodegenerative diseases. Firstly, polyphenols are derived from natural plants and can be obtained in daily diet. They have fewer side effects and are suitable for long-term use. Secondly, polyphenols can treat neurodegenerative diseases through multiple targets, which is undoubtedly crucial for the therapy of heterogeneous diseases. However, polyphenols are currently rarely used as drugs for the treatment of neurodegenerative diseases. Future large-scale multi-center randomized controlled trials and in-depth mechanism studies are still needed to fully evaluate the safety, effectiveness and possible side effects of polyphenols as therapeutic agents.

## Author contributions

All authors listed have made a substantial, direct, and intellectual contribution to the work and approved it for publication.

## References

[1] Perl DP. Neuropathology of Alzheimer's disease. Mt Sinai J Med. (2010) 77:32–42. 10.1002/msj.20157 20101720 PMC2918894

[2] Selkoe DJ Hardy J. The amyloid hypothesis of Alzheimer's disease at 25 years. EMBO Mol Med. (2016) 8:595–608. 10.15252/emmm.201606210 27025652 PMC4888851

[3] Masliah E Mallory M Alford M DeTeresa R Hansen LA McKeel DW. Altered expression of synaptic proteins occurs early during progression of Alzheimer's disease *Neurology*. (2001) 56:127–9. 10.1212/WNL.56.1.127 11148253

[4] Scheff SW Price DA Schmitt FA DeKosky ST Mufson EJ. Synaptic alterations in Ca1 in mild alzheimer disease and mild cognitive impairment. Neurology. (2007) 68:1501–8. 10.1212/01.wnl.0000260698.46517.8f 17470753

[5] Shankar GM Walsh DM. Alzheimer's disease: synaptic dysfunction and abeta. Mol Neurodegener. (2009) 4:48. 10.1186/1750-1326-4-48 19930651 PMC2788538

[6] Gong Y Chang L Viola KL Lacor PN Lambert MP Finch CE. Alzheimer's disease-affected brain: presence of oligomeric a beta ligands (addls) suggests a molecular basis for reversible memory loss. Proc Natl Acad Sci U S A. (2003) 100:10417–22. 10.1073/pnas.1834302100 12925731 PMC193576

[7] Peineau S Taghibiglou C Bradley C Wong TP Liu L Lu J. Ltp inhibits Ltd in the hippocampus via regulation of Gsk3beta. Neuron. (2007) 53:703–17. 10.1016/j.neuron.2007.01.029 17329210

[8] Manach C Scalbert A Morand C Rémésy C Jiménez L. Polyphenols: food sources and bioavailability. Am J Clin Nutr. (2004) 79:727–47. 10.1093/ajcn/79.5.727 15113710

[9] Neveu V Perez-Jiménez J Vos F Crespy V du Chaffaut L Mennen L. Phenol-explorer: an online comprehensive database on polyphenol contents in foods. Database. (2010) 2010:bap024. 10.1093/database/bap024 20428313 PMC2860900

[10] Kim K-H Tsao R Yang R Cui SW. Phenolic acid profiles and antioxidant activities of wheat bran extracts and the effect of hydrolysis conditions. Food Chem. (2006) 95:466–73. 10.1016/j.foodchem.2005.01.032

[11] Adom KK Liu RH. Antioxidant activity of grains. J Agric Food Chem. (2002) 50:6182–7. 10.1021/jf0205099 12358499

[12] Chandrasekara A Shahidi F. Content of insoluble bound phenolics in millets and their contribution to antioxidant capacity. J Agric Food Chem. (2010) 58:6706–14. 10.1021/jf100868b 20465288

[13] Pandey KB Rizvi SI. Plant polyphenols as dietary antioxidants in human health and disease. Oxid Med Cell Longev. (2009) 2:270–8. 10.4161/oxim.2.5.9498 20716914 PMC2835915

[14] Tsao R. Chemistry and biochemistry of dietary polyphenols. Nutrients. (2010) 2:1231–46. 10.3390/nu2121231 22254006 PMC3257627

[15] Rana A Samtiya M Dhewa T Mishra V Aluko RE. Health benefits of polyphenols: a concise review. J Food Biochem. (2022) 46:e14264. 10.1111/jfbc.14264 35694805

[16] Rountree SD Chan W Pavlik VN Darby EJ Siddiqui S Doody RS. Persistent treatment with cholinesterase inhibitors and/or memantine slows clinical progression of Alzheimer disease. Alzheimers Res Ther. (2009) 1:7. 10.1186/alzrt7 19845950 PMC2874259

[17] Cheng YC Sheen JM Hu WL Hung YC. Polyphenols and oxidative stress in atherosclerosis-related ischemic heart disease and stroke. Oxid Med Cell Longev. (2017) 2017:8526438. 10.1155/2017/8526438 29317985 PMC5727797

[18] Potì F Santi D Spaggiari G Zimetti F Zanotti I. Polyphenol health effects on cardiovascular and neurodegenerative disorders: a review and meta-analysis. Int J Mol Sci. (2019) 20:351. 10.3390/ijms20020351 30654461 PMC6359281

[19] Griñán-Ferré C Bellver-Sanchis A Izquierdo V Corpas R Roig-Soriano J Chillón M. The pleiotropic neuroprotective effects of resveratrol in cognitive decline and Alzheimer's disease pathology: from antioxidant to epigenetic therapy. Ageing Res Rev. (2021) 67:101271. 10.1016/j.arr.2021.101271 33571701

[20] Aryal S Skinner T Bridges B Weber JT. The pathology of Parkinson's disease and potential benefit of dietary polyphenols. Molecules. (2020) 25:19. 10.3390/molecules25194382 32987656 PMC7582699

[21] Lin K Zhou M Leng C Tao X Zhou R Li Y. Neuroprotective effect of polyphenol extracts from terminalia chebula retz. against cerebral ischemia-reperfusion injury. Molecules. (2022) 27:449. 10.3390/molecules27196449 36234986 PMC9571999

[22] Yang Y He B Zhang X Yang R Xia X Chen L. Geraniin protects against cerebral ischemia/reperfusion injury by suppressing oxidative stress and neuronal apoptosis via regulation of the Nrf2/Ho-1 Pathway. Oxid Med Cell Longev. (2022) 2022:2152746. 10.1155/2022/2152746 35222793 PMC8881129

[23] Ye M Wu H Li S. Resveratrol alleviates oxygen/glucose deprivation/reoxygenation-induced neuronal damage through induction of mitophagy. Mol Med Rep. (2021) 23:11. 10.3892/mmr.2020.11711 33236158 PMC7716397

[24] Shi N Zhu C Li L. Rehabilitation training and resveratrol improve the recovery of neurological and motor function in rats after cerebral ischemic injury through the sirt1 signaling pathway. Biomed Res Int. (2016) 2016:1732163. 10.1155/2016/1732163 28116292 PMC5223001

[25] Zaidi FK Deep S. Scutellarin inhibits the uninduced and metal-induced aggregation of α-synuclein and disaggregates preformed fibrils: implications for Parkinson's disease. Biochem J. (2020) 477:645–70. 10.1042/BCJ20190705 31939603

[26] Sharma N Soni R Sharma M Chatterjee S Parihar N Mukarram M. Chlorogenic acid: a polyphenol from coffee rendered neuroprotection against rotenone-induced Parkinson's disease by Glp-1 secretion. Mol Neurobiol. (2022) 59:6834–56. 10.1007/s12035-022-03005-z 36048341

[27] Chen C Wei YZ He XM Li DD Wang GQ Li JJ. Naringenin produces neuroprotection against lps-induced dopamine neurotoxicity via the inhibition of microglial Nlrp3 inflammasome activation. Front Immunol. (2019) 10:936. 10.3389/fimmu.2019.00936 31118933 PMC6504827

[28] Parekh P Sharma N Sharma M Gadepalli A Sayyed AA Chatterjee S. Ampk-dependent autophagy activation and alpha-synuclein clearance: a putative mechanism behind alpha-mangostin's neuroprotection in a rotenone-induced mouse model of Parkinson's disease. Metab Brain Dis. (2022) 37:2853–70. 10.1007/s11011-022-01087-1 36178640

[29] Wang QQ Gao H Yuan R Han S Li XX Tang M. et al. Procyanidin A2, a polyphenolic compound, exerts anti-inflammatory and anti-oxidative activity in lipopolysaccharide-stimulated Raw2647 cells. PLoS ONE. (2020) 15:e0237017. 10.1371/journal.pone.0237017 32756588 PMC7406031

[30] Hu X Liu C Wang K Zhao L Qiu Y Chen H. Multifunctional anti-Alzheimer's disease effects of natural xanthone derivatives: a primary structure-activity evaluation. Front Chem. (2022) 10:842208. 10.3389/fchem.2022.842208 35646819 PMC9130743

[31] Chen Y Bian Y Wang JW Gong TT Ying YM Ma LF. Effects of α-mangostin derivatives on the Alzheimer's disease model of rats and their mechanism: a combination of experimental study and computational systems pharmacology analysis. ACS Omega. (2020) 5:9846–63. 10.1021/acsomega.0c00057 32391472 PMC7203693

[32] De Lorenzi E Franceschini D Contardi C Di Martino RMC Seghetti F Serra M. Modulation of amyloid α-induced microglia activation and neuronal cell death by curcumin and analogues. Int J Mol Sci. (2022) 23:81. 10.3390/ijms23084381 35457197 PMC9027876

[33] Pupyshev AB Belichenko VM Tenditnik MV Bashirzade AA Dubrovina NI Ovsyukova MV. Combined induction of mtor-dependent and mtor-independent pathways of autophagy activation as an experimental therapy for Alzheimer's disease-like pathology in a mouse model. Pharmacol Biochem Behav. (2022) 217:173406. 10.1016/j.pbb.2022.173406 35609863

[34] Wang H Jiang T Li W Gao N Zhang T. Resveratrol attenuates oxidative damage through activating mitophagy in an in vitro model of Alzheimer's disease. Toxicol Lett. (2018) 282:100–8. 10.1016/j.toxlet.2017.10.021 29097221

[35] Barthels D Das H. Current advances in ischemic stroke research and therapies. Biochim Biophys Acta Mol Basis Dis. (2020) 1866:165260. 10.1016/j.bbadis.2018.09.012 31699365 PMC6981280

[36] Liu J He J Huang Y Hu Z. Resveratrol has an overall neuroprotective role in ischemic stroke: a meta-analysis in rodents. Front Pharmacol. (2021) 12:795409. 10.3389/fphar.2021.795409 34987407 PMC8721173

[37] Wu MY Yiang GT Liao WT Tsai AP Cheng YL Cheng PW. Current mechanistic concepts in ischemia and reperfusion injury. Cell Physiol Biochem. (2018) 46:1650–67. 10.1159/000489241 29694958

[38] Caprio FZ Sorond FA. Cerebrovascular disease: primary and secondary stroke prevention. Med Clin North Am. (2019) 103:295–308. 10.1016/j.mcna.2018.10.001 30704682

[39] Liao S Apaijai N Chattipakorn N Chattipakorn SC. The possible roles of necroptosis during cerebral ischemia and ischemia / reperfusion injury. Arch Biochem Biophys. (2020) 695:108629. 10.1016/j.abb.2020.108629 33068524

[40] Gasparovic AC Zarkovic N Bottari SP. Biomarkers of nitro-oxidation and oxidative stress. Current Opinion Toxicol. (2018) 7:73–80. 10.1016/j.cotox.2017.10.002

[41] Jurcau A Ardelean AI. Oxidative stress in ischemia/reperfusion injuries following acute ischemic stroke. Biomedicines. (2022) 10:3. 10.3390/biomedicines10030574 35327376 PMC8945353

[42] Rodrigo R Fernández-Gajardo R Gutiérrez R Matamala JM Carrasco R Miranda-Merchak A. Oxidative stress and pathophysiology of ischemic stroke: novel therapeutic opportunities. CNS Neurol Disord Drug Targets. (2013) 12:698–714. 10.2174/1871527311312050015 23469845

[43] Celotti E Ferrarini R Zironi R Conte LS. Resveratrol content of some wines obtained from dried valpolicella grapes: recioto and amarone. J Chromatogr A. (1996) 730:47–52. 10.1016/0021-9673(95)00962-0 8680595

[44] Pany S Majhi A Das J. Pkc Activation by Resveratrol Derivatives with Unsaturated Aliphatic Chain. PLoS One. (2012) 7:e52888. 10.1371/journal.pone.0052888 23285216 PMC3528653

[45] Das J Pany S Majhi A. Chemical modifications of resveratrol for improved protein kinase C alpha activity. Bioorg Med Chem. (2011) 19:5321–33. 10.1016/j.bmc.2011.08.008 21880495

[46] Wang EH Yu ZL Ping GF Zhai S. Grape seed procyanidin extract attenuate sodium fluoride-induced oxidative damage and apoptosis in rat kidneys. Biomed Environ Sci. (2020) 33:454–7. 10.3967/bes2020.061 32641209

[47] Zeng YX Wang S Wei L Cui YY Chen YH. Proanthocyanidins: components, pharmacokinetics and biomedical properties. Am J Chin Med. (2020) 48:813–69. 10.1142/S0192415X2050041X 32536248

[48] Safwen K Selima S Mohamed E Ferid L Pascal C Mohamed A. Protective effect of grape seed and skin extract on cerebral ischemia in rat: implication of transition metals. Int J Stroke. (2015) 10:415–24. 10.1111/ijs.12391 25365917

[49] Trudler D Nash Y Frenkel D. New insights on Parkinson's disease genes: the link between mitochondria impairment and neuroinflammation. J Neural Transm. (2015) 122:1409–19. 10.1007/s00702-015-1399-z 25894287

[50] Dong AQ Yang YP Jiang SM Yao XY Qi D Mao CJ. Pramipexole inhibits astrocytic nlrp3 inflammasome activation via Drd3-dependent autophagy in a mouse model of Parkinson's disease. Acta Pharmacol Sin. (2022). 10.1038/s41401-022-00951-1 35896696 PMC9813225

[51] Cheng X-Y Mao C-J Wang Y-L Liu C-F. Gastrointestinal symptoms of Parkinson's disease: a systematic review from pathogenesis to management. Adv Neurol. (2022) 1:1–16. 10.36922/an.v1i1.9

[52] Simon DK Tanner CM Brundin P. Parkinson disease epidemiology, pathology, genetics, and pathophysiology. Clin Geriatr Med. (2020) 36:1–12. 10.1016/j.cger.2019.08.002 31733690 PMC6905381

[53] Fathi M Vakili K Yaghoobpoor S Qadirifard MS Kosari M Naghsh N. Pre-Clinical studies identifying molecular pathways of neuroinflammation in Parkinson's disease: a systematic review. Front Aging Neurosci. (2022) 14:855776. 10.3389/fnagi.2022.855776 35912090 PMC9327618

[54] Kempuraj D Thangavel R Fattal R Pattani S Yang E Zaheer S. Mast cells release chemokine Ccl_2_ in response to parkinsonian toxin 1-methyl-4-phenyl-pyridinium (Mpp(+)). Neurochem Res. (2016) 41:1042–9. 10.1007/s11064-015-1790-z 26646004 PMC4834226

[55] Lücking CB Dürr A Bonifati V Vaughan J De Michele G Gasser T. Association between early-onset Parkinson's disease and mutations in the parkin gene. N Engl J Med. (2000) 342:1560–7. 10.1056/NEJM200005253422103 10824074

[56] Kitada T Asakawa S Hattori N Matsumine H Yamamura Y Minoshima S. Mutations in the parkin gene cause autosomal recessive juvenile parkinsonism. Nature. (1998) 392:605–8. 10.1038/33416 9560156

[57] Wasner K Smajic S Ghelfi J Delcambre S Prada-Medina CA Knappe E. Parkin deficiency impairs mitochondrial DNA dynamics and propagates inflammation. Mov Disord. (2022) 37:1405–15. 10.1002/mds.29025 35460111

[58] Lastres-Becker I Ulusoy A Innamorato NG Sahin G Rábano A Kirik D. α-Synuclein expression and Nrf2 deficiency cooperate to aggravate protein aggregation, neuronal death and inflammation in early-stage Parkinson's Disease. Hum Mol Genet. (2012) 21:3173–92. 10.1093/hmg/dds143 22513881

[59] Lei K Shen Y He Y Zhang L Zhang J Tong W. Baicalin represses C/Ebpβ via its antioxidative effect in Parkinson's disease. Oxid Med Cell Longev. (2020) 2020:8951907. 10.1155/2020/8951907 32566108 PMC7261332

[60] Fan Y Li Y Yang Y Lin K Lin Q Luo S. Chlorogenic acid prevents microglia-induced neuronal apoptosis and oxidative stress under hypoxia-ischemia environment by regulating the Mir497hg/Mir-29b-3p/Sirt1 axis. Dis Markers. (2022) 2022:1194742. 10.1155/2022/1194742 35664431 PMC9159818

[61] Wu Y Hu Y Wang B Li S Ma C Liu X. Dopamine uses the Drd5-Arrb2-Pp2a signaling axis to block the Traf6-mediated Nf-Kb pathway and suppress systemic inflammation. Mol Cell. (2020) 78:42–56. 10.1016/j.molcel.2020.01.022 32035036

[62] Shao W Zhang SZ Tang M Zhang XH Zhou Z Yin YQ. Suppression of neuroinflammation by astrocytic dopamine D2 receptors via αb-crystallin. Nature. (2013) 494:90–4. 10.1038/nature11748 23242137

[63] Ransohoff RM. How neuroinflammation contributes to neurodegeneration. Science. (2016) 353:777–83. 10.1126/science.aag2590 27540165

[64] Hirsch EC Hunot S. Neuroinflammation in Parkinson's disease: a target for neuroprotection? Lancet Neurol. (2009) 8:382–97. 10.1016/S1474-4422(09)70062-6 19296921

[65] Labzin LI Heneka MT Latz E. Innate immunity and neurodegeneration. Annu Rev Med. (2018) 69:437–49. 10.1146/annurev-med-050715-104343 29106805

[66] Guan Y Han F. Key Mechanisms and potential targets of the Nlrp3 inflammasome in neurodegenerative diseases. Front Integr Neurosci. (2020) 14:37. 10.3389/fnint.2020.00037 32792920 PMC7393579

[67] Li Y Liu T Li Y Han D Hong J Yang N. Baicalin ameliorates cognitive impairment and protects microglia from lps-induced neuroinflammation via the Sirt1/Hmgb1 pathway. Oxid Med Cell Longev. (2020) 2020:4751349. 10.1155/2020/4751349 33029280 PMC7527898

[68] Ahmad MH Fatima M Ali M Rizvi MA Mondal AC. Naringenin alleviates paraquat-induced dopaminergic neuronal loss in Sh-Sy5y cells and a rat model of Parkinson's disease. Neuropharmacology. (2021) 201:108831. 10.1016/j.neuropharm.2021.108831 34655599

[69] García-Blanco A Baquero M Vento M Gil E Bataller L Cháfer-Pericás C. Potential oxidative stress biomarkers of mild cognitive impairment due to alzheimer disease. J Neurol Sci. (2017) 373:295–302. 10.1016/j.jns.2017.01.020 28131209

[70] Li NM Liu KF Qiu YJ Zhang HH Nakanishi H Qing H. Mutations of beta-amyloid precursor protein alter the consequence of Alzheimer's disease pathogenesis. Neural Regen Res. (2019) 14:658–65. 10.4103/1673-5374.247469 30632506 PMC6352587

[71] Zheng W Su Z Liu X Zhang H Han Y Song H. Modulation of functional activity and connectivity by acupuncture in patients with alzheimer disease as measured by resting-state Fmri. PLoS ONE. (2018) 13:e0196933. 10.1371/journal.pone.0196933 29763448 PMC5953467

[72] Cacace R Sleegers K Van Broeckhoven C. Molecular genetics of early-onset Alzheimer's disease revisited. Alzheimers Dement. (2016) 12:733–48. 10.1016/j.jalz.2016.01.012 27016693

[73] Orr ME Sullivan AC Frost B. A.brief overview of tauopathy: causes, consequences, and therapeutic strategies. Trends Pharmacol Sci. (2017) 38:637–48. 10.1016/j.tips.2017.03.011 28455089 PMC5476494

[74] Femminella GD Dani M Wood M Fan Z Calsolaro V Atkinson R. Microglial activation in early alzheimer trajectory is associated with higher gray matter volume. Neurology. (2019) 92:e1331–43. 10.1212/WNL.0000000000007133 30796139 PMC6511099

[75] Wasilewski D Villalba-Moreno ND Stange I Glatzel M Sepulveda-Falla D Krasemann S. Reactive astrocytes contribute to Alzheimer's disease-related neurotoxicity and synaptotoxicity in a neuron-astrocyte co-culture assay. Front Cell Neurosci. (2021) 15:739411. 10.3389/fncel.2021.739411 35126055 PMC8813976

[76] Potter KA Buck AC Self WK Callanan ME Sunil S Capadona JR. The effect of resveratrol on neurodegeneration and blood brain barrier stability surrounding intracortical microelectrodes. Biomaterials. (2013) 34:7001–15. 10.1016/j.biomaterials.2013.05.035 23791503

[77] Wang F Cui N Yang L Shi L Li Q Zhang G. Resveratrol rescues the impairments of hippocampal neurons stimulated by microglial over-activation in vitro. Cell Mol Neurobiol. (2015) 35:1003–15. 10.1007/s10571-015-0195-5 25898934 PMC11486292

[78] Zhao H Wang Q Cheng X Li X Li N Liu T. inhibitive effect of resveratrol on the inflammation in cultured astrocytes and microglia induced by Aβ(1-42). Neuroscience. (2018) 379:390–404. 10.1016/j.neuroscience.2018.03.047 29627302

[79] Hanisch UK Kettenmann H. Microglia: active sensor and versatile effector cells in the normal and pathologic brain. Nat Neurosci. (2007) 10:1387–94. 10.1038/nn1997 17965659

[80] Parkhurst CN Yang G Ninan I Savas JN Yates JR 3rd Lafaille JJ. Microglia promote learning-dependent synapse formation through brain-derived neurotrophic factor. Cell. (2013) 155:1596–609. 10.1016/j.cell.2013.11.030 24360280 PMC4033691

[81] Hickman SE Allison EK El Khoury J. Microglial dysfunction and defective beta-amyloid clearance pathways in aging Alzheimer's disease mice. J Neurosci. (2008) 28:8354–60. 10.1523/JNEUROSCI.0616-08.2008 18701698 PMC2597474

[82] Doens D Fernández PL. Microglia receptors and their implications in the response to amyloid β for Alzheimer's disease pathogenesis. J Neuroinflammation. (2014) 11:48. 10.1186/1742-2094-11-48 24625061 PMC3975152

[83] Pulido-Moran M Moreno-Fernandez J Ramirez-Tortosa C Ramirez-Tortosa M. Curcumin and health. Molecules. (2016) 21:264. 10.3390/molecules21030264 26927041 PMC6273481

[84] Braak H Braak E. Morphological criteria for the recognition of Alzheimer's disease and the distribution pattern of cortical changes related to this disorder. Neurobiol Aging. (1994) 15:355–6. 10.1016/0197-4580(94)90032-9 7936061

[85] Heneka MT Carson MJ El Khoury J Landreth GE Brosseron F Feinstein DL. Neuroinflammation in Alzheimer's disease. Lancet Neurol. (2015) 14:388–405. 10.1016/S1474-4422(15)70016-5 25792098 PMC5909703

[86] Varnum MM Ikezu T. The classification of microglial activation phenotypes on neurodegeneration and regeneration in Alzheimer's disease brain. Arch Immunol Ther Exp. (2012) 60:251–66. 10.1007/s00005-012-0181-2 22710659 PMC4429536

[87] Fang X Zhang J Zhao J Wang L. Effect of resveratrol combined with donepezil hydrochloride on inflammatory factor level and cognitive function level of patients with Alzheimer's disease. J Healthc Eng. (2022) 2022:9148650. 10.1155/2022/9148650 35368930 PMC8975642

